# Frequency of Autonomic Dysfunction in Patients of Guillain Barre Syndrome in a Tertiary Care Hospital

**DOI:** 10.7759/cureus.12101

**Published:** 2020-12-15

**Authors:** Jay Singh, Vekash Raja, Muhammad Irfan, Owais Hashmat, Mohsina Syed, Naila N Shahbaz

**Affiliations:** 1 Neurology, Dow University of Health Sciences, Civil Hospital, Karachi, PAK; 2 Neurology, Jinnah Postgraduate Medical Centre, Karachi, PAK; 3 Neurology, Aga Khan University, Karachi, PAK

**Keywords:** autonomic dysfunction, dysautonomia, hypertension, guillain-barré syndrome, acute inflammatory demyelinating polyradiculoneuropathy

## Abstract

Introduction

Guillain-Barré syndrome (GBS) is defined as a syndrome manifesting as an acute inflammatory demyelinating polyradiculoneuropathy (AIDP) with coexistent weakness and absent or diminished reflexes clinically.Autonomic dysfunction (AD) or dysautonomia is a common finding in GBS. Autonomic dysfunction usually occurs in the acute phase of the illness but can also be seen in the recovery phase. The rationale of our study is to determine the frequency of autonomic dysfunction in patients of GBS admitted to the Neurology department of Civil Hospital, Karachi.

Methods

A total of 118 admitted patients at a tertiary care hospital in Pakistan who fulfilled the inclusion criteria were enrolled in the study after informed consent. The study was conducted for six months at the department of neurology, Civil Hospital, Karachi. Patients were assessed for autonomic dysfunction by recording blood pressures and pulse rate hourly (both lying and standing positions) by resident doctors. Urinary retention, diarrhea, and constipation were also recorded in a separate chart. All values entered in the pre-approved performa by researchers. The data was collected and analyzed on Statistical Package for Social Sciences (SPSS) version 18.0 (IBM Corp., Armonk, NY, USA). Descriptive statistics included mean, standard deviation (SD) of continuous data, like age, duration of illness, motor weakness assessment by Medical Research Council (MRC) Scale, protein content in cerebrospinal fluid (CSF), pulse, and blood pressure at the time of presentation. Frequencies and percentages were calculated from the categorical data, like gender and patients with autonomic dysfunction (outcome variable). Effect modifiers were controlled by stratification of age, gender, duration of illness. Post-stratification chi-square test was applied with a p-value of ≤ 0.05 taken as significant.

Results

In our study, the average age of the patients was 39.90±9.91 years. Frequency of autonomic dysfunction among patients with GBS was 41.53% (49/118). The most frequent autonomic manifestations were constipation and diarrhea; 22% and 21.2% respectively. Additional manifestations included urinary retention (15.3%) and fluctuation of blood pressure and heart rate at 13.6% each.

Conclusion

This study showed that the frequency of autonomic dysfunction among patients of Guillain Barre Syndrome was significant, consistent with previous studies. Our study explored the adverse outcomes of autonomic dysfunction in patients with GBS. This will help physicians increase their understanding of dysautonomia so that effective management plans can be formulated for patients with GBS to prevent adverse outcomes and hence provide better patient care.

## Introduction

Guillain-Barré syndrome (GBS) is described as a syndrome manifesting clinically as an acute inflammatory polyradiculoneuropathy (AIDP) with concomitant, symmetrical muscular weakness with absent or diminished deep tendon reflexes [1]. Patients usually present a few days to a week after onset of symptoms. The weakness can vary from mild difficulty with walking to nearly complete paralysis of all extremity, respiratory, and bulbar muscles. It has been reported worldwide [2,3], with an incidence of one to two cases per 100,000 per year [4]. The incidence increases 20% for every 10-year increase in age and males are more at risk than females [4].

It is widely thought that GBS results from an immune response to a preceding infection that cross-reacts with peripheral nerve components involving molecular mimicry. The immune response can be directed towards the myelin or the axon of the peripheral nerve, resulting in demyelinating or axonal forms of GBS.

In literature, *Campylobacter jejuni* infection is the most commonly identified precipitant of GBS. Cytomegalovirus, Epstein-Barr virus, human immunodeficiency virus (HIV), and Zika virus have also been reported with GBS [5]. A small percentage of patients develop GBS after other triggering events such as immunization, surgery, trauma, and bone-marrow transplantation.

The initial diagnosis of GBS is based upon the clinical presentation. The clinical diagnosis is supportive if cerebrospinal fluid (CSF) and electrodiagnostic studies show typical abnormalities [6]. Therefore, lumbar puncture and electrodiagnostic studies are performed, irrespective of clinical presentation, in all patients with suspected GBS. Therefore, these tests are also helpful to clinicians in excluding alternative diagnoses.

The clinical presentation of GBS is variable with weakness usually starting in the legs, but earlier involvement of arms or facial muscles is reported in about 10% of patients. Patients may require ventilator support in case of respiratory muscle weakness in 10 to 30% of patients [7]. Other features include facial and oculomotor nerve involvement, areflexia, or decreased reflexes with paresthesias and pain during the acute phase of the illness.

Autonomic dysfunction (AD) in GBS predominantly occurs in the acute phase of the illness but can manifest in the recovery phase too. The exact mechanism remains unknown but probably involves dysfunction in the sympathetic and parasympathetic systems [8].

AD in GBS is observed in 70% of the cases [7]; features including tachycardia, bradycardia, facial flushing, hypertension alternating with hypotension, orthostatic hypotension, anhydrosis or diaphoresis, and urinary retention while gastrointestinal autonomic manifestation includes diarrhea or constipation [9]. Severe autonomic dysfunction is an important factor to recognize and treat accordingly as this is occasionally associated with a sudden death rate of 5-7% [10,11].

Therefore, it is evident that autonomic dysfunction in GBS needs to be identified and warrants urgent medical treatment owing to its high prevalence and also because it counts for a relatively high sudden death rate.

## Materials and methods

We conducted a six-month cross-sectional study of consenting patients admitted at the Department of Neurology in Civil Hospital, Karachi, Pakistan with a diagnosis of GBS. Inclusion criteria included all patients of either sex aged 18 to 70 years admitted with diagnosis of GBS not received any treatment for GBS. Non-consenting patients were excluded from the study along with those patients with past history of GBS, Glasgow Coma Scale score < 8, and previous history of stroke or head trauma. Patients on medications such as corticosteroids were also excluded. A brief history of the duration of illness and demographic information was taken from each patient and confirmed by an attendant. Patients were assessed for AD by taking hourly measurements of blood pressure and pulse rate by resident doctors in lying as well as standing positions. Urinary retention, diarrhea, and constipation were recorded in a separate intake output chart. All findings were entered into a pre-approved proforma.

A total of 118 patients of GBS fulfilling the inclusion criteria were identified and included in the study after informed consent. Patients were diagnosed with GBS on clinical examination [6], absence of deep tendon reflexes; in the presence of all of three features: 1) motor weakness: decreased muscle power (Grade <2), present for >four weeks as assessed by the Medical Research Council (MRC) scale, Grade 5: normal power, Grade 4: active movement of limbs against gravity upon resistance of one fist, Grade 3: active movement of limbs against gravity without any resistance, Grade 2: active movement of limbs both laterally and medially without any resistance, Grade 1: only flicker or contraction present, and Grade 0: no movement of limbs; 2) cerebrospinal fluid (CSF) analysis: presence of CSF protein > 55mg/dl without an increase in white blood cells; 3) electromyography and nerve conduction velocities: findings of demyelination: absence of F waves and H reflexes. Autonomic dysfunction (AD) defined as by the presence of any two of the following: 1) fluctuating (labile) blood pressure: >20% variation in blood pressure (checked in lying and standing positions, both rise and fall in variations noted) more than two times in 24 hours when checked on intervals of one hour; 2) fluctuating (labile) heart rate: > 10% variation in heart rate (both rise and fall) more than two times in 24 hours when checked on intervals of one hour; 3) urinary retention: no passage of urine for >12 hours with filled urinary bladder detected on ultrasound despite intake of minimum 1500 ml of oral fluids; 4) gastrointestinal autonomic dysfunction: presence of diarrhea (passage of stools >three times in 24 hours) or constipation (passage of stools <two times in 48 hours).

Data were analyzed using Statistical Package for Social Sciences (SPSS) version 18.0 (IBM Corp., Armonk, NY, USA). Descriptive statistics included mean ± standard deviation (SD) of continuous data, like age, duration of GBS, MRC grade of muscle power, protein in CSF, pulse and blood pressure at the time of presentation and during admission. Frequencies and percentages were calculated from the categorical data, like gender and patients with autonomic dysfunction (outcome variable). Effect modifiers were controlled by stratification of age, gender, and duration of GBS (< four weeks). Post-stratification chi-square test was applied with a p-value of ≤ 0.05 taken as significant. We applied non-probability consecutive sampling. For sample size calculation, we used OpenEpi, taking confidence level (1-α) of 95% and anticipated population proportion (P) of 3.9% [10] with absolute precision (d) of 3.5%, the sample size of the study calculated was 118.

 Z2 x (P) x (1-P)

 _____________

 d2

Where Z = Z value (e.g. 1.96 for 95% confidence interval level), P = prevalence of disease, d = absolute precision of 3.5% (0.035).

## Results

**Figure 1:**
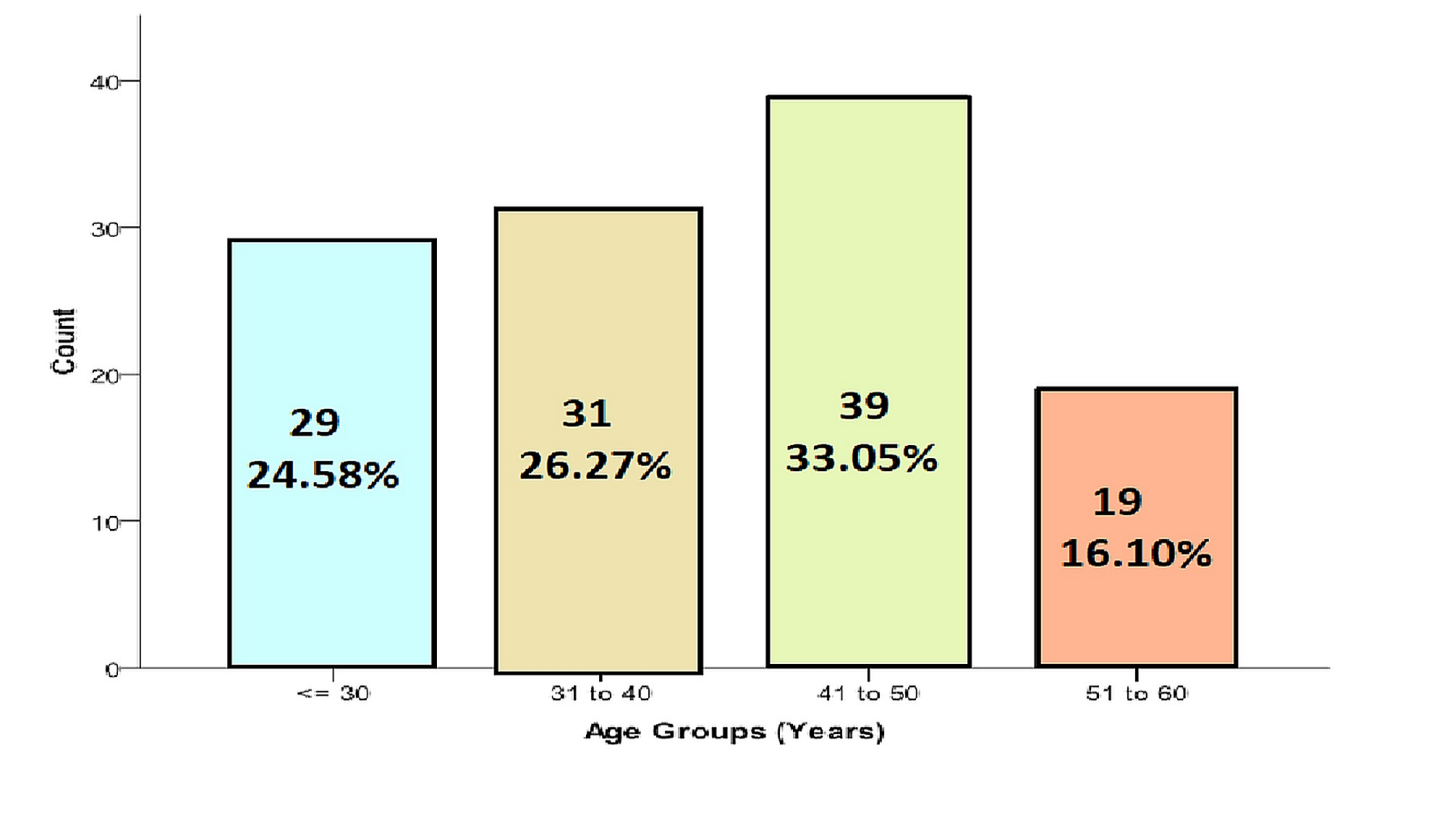
Age distribution of the patients (n=118)

**Figure 2:**
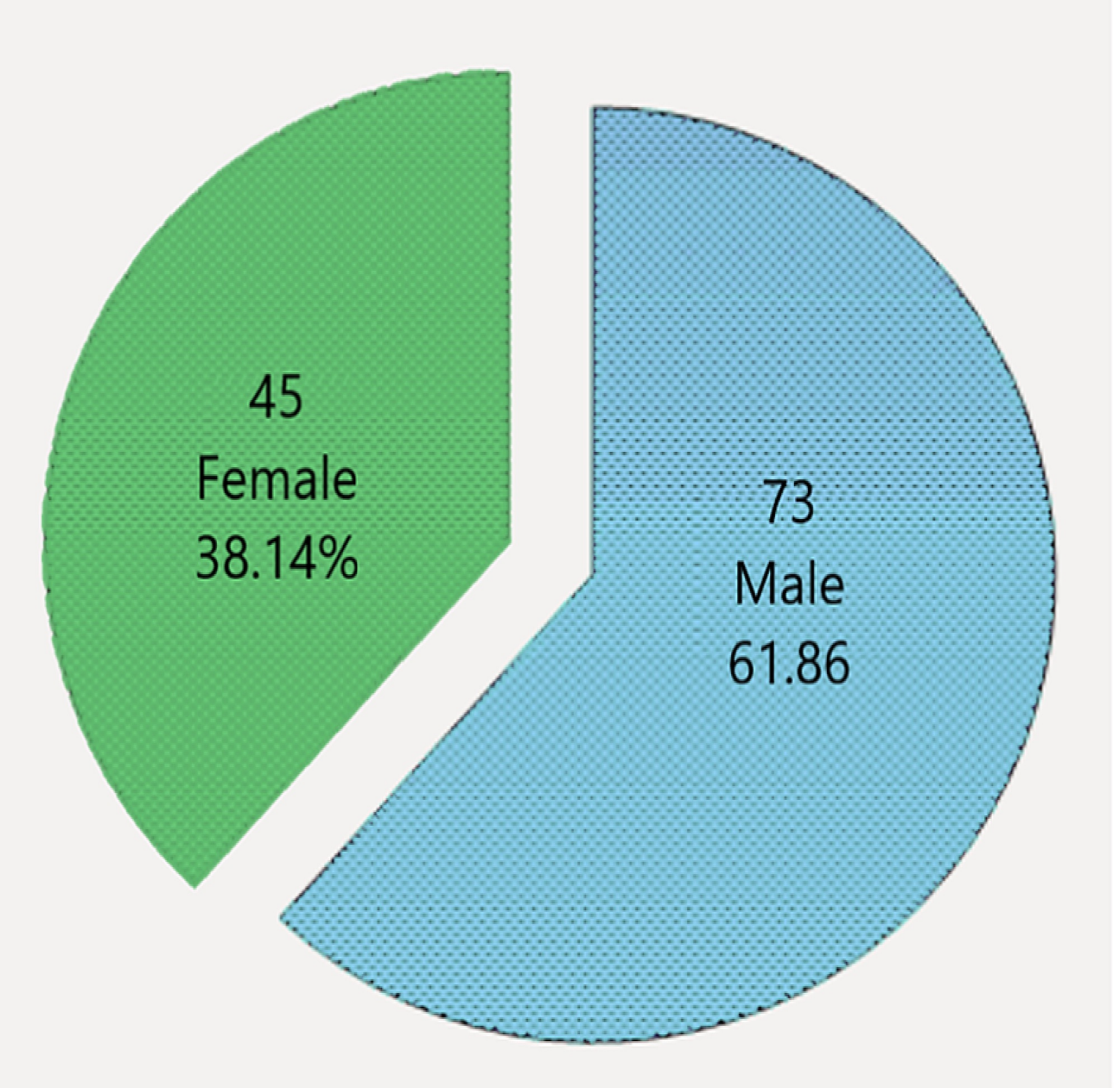
Gender distribution of the patients (n=118)

A total of 118 patients with GBS were included in the study according to the criteria of the protocol. The average age of the patients was 39.90±9.91 years (Figure 1). There were 73 (61.86%) males and 45 (38.14%) females (Figure 2).

**Figure 3:**
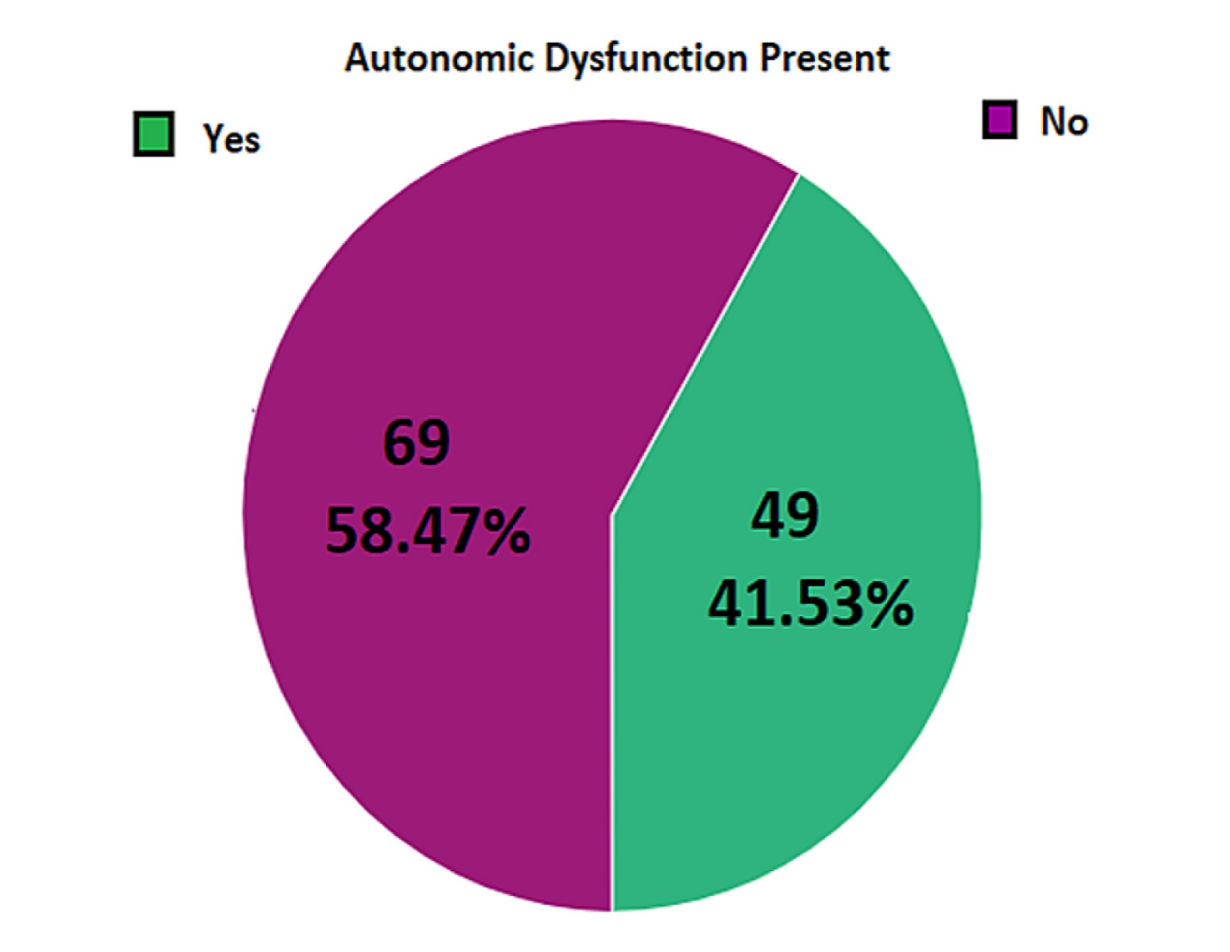
Frequency of autonomic dysfunction among the patients of Guillain-Barré syndrome (n=118)

Frequency of autonomic dysfunction among patients of GBS observed was 41.53% (49/118) as presented in Figure 3.

Most frequent manifestations were constipation and diarrhea at 22% and 21.2%, respectively; urinary retention was 15.3% and fluctuation of blood pressure and heart rate was 13.6% each as shown in Table 1 and Table 2. 

**Table 1:** Descriptive statistics of characteristics of patients (n=118) Abbreviations: GBS, Guillain-Barré syndrome; MRC, Medical Research Council; CSF, cerebrospinal fluid; SBP, systolic blood pressure; DBP, diastolic blood pressure

Variables	Mean	Standard Deviation	95% Confidence Interval for Mean	
			Lower Bound	Upper Bound
Age (Years)	39.9	9.91	38.09	41.7
Duration of GBS (Weeks)	5.77	1.41	5.51	6.03
MRC Muscle Power	1.77	0.54	1.67	1.87
Protein in CSF (mg)	63.75	3.95	63.03	64.48
Pulse (beats/min)	86.28	6.49	85.1	87.46
SBP (mm Hg)	131	8.04	129.53	132.47
DBP (mm Hg)	86.3	6.07	85.19	87.4

**Table 2:** Types of autonomic dysfunction among patients of Guillain-Barré syndrome (n=118) Count is more than 49 because of multiple dysfunction

Autonomic Dysfunction	Frequency	Percentage
Fluctuation in Blood Pressure	23	13.6%
Fluctuation in Heart Rate	16	13.6%
Urinary Retention	18	15.3%
Diarrhea	25	21.2%
Constipation	26	22%
Fluctuating (Labile) Blood Pressure :> 20% variation in Blood Pressure (both rise and fall) more than two times in 24 hours when checked on interval of 1 hour. Fluctuating (Labile) Heart Rate :> 10% variation in heart rate (both rise and fall) more than two times in 24 hours when checked on interval of 1 hour. Urinary Retention: No passage of Urine for >12 hours with filled urinary bladder detected on ultrasound despite intake of minimum1500 ml of oral fluids Gastrointestinal autonomic dysfunction: presence of diarrhea (passage of stools > three times in 24 hours) or constipation (passage of stools < two times in 48 hours		

Stratification analysis was performed and observed that overall rate of autonomic dysfunction was not significant among different age groups (p=0.092) while in fluctuation in BP was significantly high in lower age groups (p=0.010) as shown in Table 3 and Table 4.

**Table 3:** Frequency of autonomic dysfunction among patients of Guillain-Barré syndrome by age groups (n=118)

Age Groups	Autonomic Dysfunction		Total	Chi-Square	P-Value
	Yes	No			
≤ 30	15(51.7%)	14(48.3%)	29	6.436	0.092
31 to 40	7(22.6%)	24(77.4%)	33		
41 to 50	18(46.2%)	21(53.8%)	21		
51 to 60	9(47.4%)	10(52.6%)	10		

**Table 4:** Types of autonomic dysfunction among patients of Guillain-Barré syndrome (n=118)

Autonomic Dysfunction	Age Groups (Years)				P-Value
	≤30 n=29	31 to 40 n=31	41 to 50 n=39	50-60 n=19	
Fluctuation in Blood Pressure	10(34.5%)	1(3.2%)	10(25.6%)	2(10.5%)	0.010
Fluctuation in Heart Rate	7(24.1%)	1(3.2%)	6(15.4%)	2(10.5%)	0.119
Urinary Retention	4(13.8%)	4(12.9%)	5(12.8%)	5(26.3%)	0.540
Diarrhea	7(24.1%)	4(12.9%)	10(25.6%)	4(21.1%)	0.596
Constipation	8(27.6%)	4(12.9%)	9(23.1%)	5(26.3%)	0.522

Rate of autonomic dysfunction was not significant between males and females as shown in Table 5 and Table 6, respectively.

**Table 5:** Frequency of autonomic dysfunction among patients of Guillain-Barré syndrome by gender distribution (n=118)

Gender	Autonomic Dysfunction		Total	Chi-Square	P-Value
	Yes	No			
Male	29(39.7%)	44(60.3%)	73	0.255	0.613
Female	20(44.4%)	25(55.6%)	45		

**Table 6:** Types of autonomic dysfunction among patients of Guillain-Barré syndrome by gender distribution (n=118)

Autonomic Dysfunction	Gender		P-Value
	Male n=73	Female n=45	
Fluctuation in Blood Pressure	13(17.8%)	10(22.2%)	0.557
Fluctuation in Heart Rate	11(15.1%)	5(11.1%)	0.542
Urinary Retention	10(13.7%)	8(17.8%)	0.549
Diarrhea	16(21.9%)	9(20%)	0.804
Constipation	16(21.9%)	10(22.2%)	0.969

Rate of autonomic dysfunction among patients of GBS was significantly high in those cases whose duration of GBS was above five weeks as compared to four to five weeks (p=0.005, Table 7).

**Table 7:** Frequency of autonomic dysfunction among patients of Guillain-Barré syndrome (GBS) by duration (n=118)

Duration of GBS (Weeks)	Autonomic Dysfunction		Total	Chi-Square	P-Value
	Yes	No			
4-5	20(30.3%)	46(69.7%)	66	7.768	0.005
>5	29(55.8%)	23(44.2%)	52		

Fluctuation in BP and diarrhea was significantly higher in those cases whose duration of GBS was above five weeks as compared to four to five weeks as shown in Table 8.

**Table 8:** Types of autonomic dysfunction among patients of Guillain-Barré syndrome (GBS) by duration (n=118)

Autonomic dysfunction	Duration of GBS		P-Value
	4 to 5 Weeks n=66	>5 weeks n=52	
Fluctuation in Blood Pressure	7(10.6%)	16(30.8%)	0.006
Fluctuation Heart Rate	9(13.6%)	7(13.5%)	0.978
Urinary Retention	7(10.6%)	11(21.2%)	0.114
Diarrhea	9(13.6%)	16(30.8%)	0.024
Constipation	11(16.7%)	15(28.8%)	0.113

## Discussion

AD is a well-recognized feature of GBS and is a significant source of mortality [12]. According to one study, AD occurs in 70% of patients and manifests as symptoms that include tachycardia (the most common), urinary retention, hypertension alternating with hypotension, orthostatic hypotension, bradycardia, other arrhythmias, ileus, and loss of sweating [8].

Severe autonomic disturbances occur in about 20% of patients, mostly (but not always) in patients who develop severe weakness and respiratory failure. Consequently, close monitoring of blood pressure, fluid status, and cardiac rhythm is essential to the management of patients with GBS [8].

Cardiovascular complications of GBS are an important cause of morbidity. Common manifestations include paroxysmal fluctuations in blood pressure, and tachyarrhythmia and bradyarrhythmias, while less frequent manifestations include myocardial involvement ranging from myocarditis to heart failure. Therefore, it is recommended to monitor cardiac rhythm and blood pressure in the progressive phase of GBS [13]. Monitoring should be instituted at time of admission and continued until ventilator support is no longer necessary or until recovery is underway.

Regarding specific autonomic dysfunctions, the largest study done by Anandan et al. included 2,587 patients and over 10,000 controls [14]. It concluded that in hospitalized patients with GBS, AD most frequently manifests as alternating diarrhea/constipation (15.5%), hyponatremia (14.9%), syndrome of inappropriate antidiuretic hormone secretion (SIADH; 4.8%), bradycardia (4.7%), and urinary retention (3.9%). Verma et al. have reported 34.4% patients with AD in GBS [15].

Previous studies in Pakistan [16,17] regarding autonomic dysfunction in GBS have not identified which specific autonomic dysfunction is more prevalent in our population. In one study, Nabi et al. found that 61.3% of mechanically ventilated patients of GBS had AD; however, no specific autonomic dysfunction was identified in studied patients [16].

So the rationale of our study was to elucidate the current magnitude of each specific autonomic dysfunction in GBS so it would be helpful in identifying early treatment of separate AD in time and reducing morbidity in GBS.

Our study had a few limitations. Our study used a smaller sample size of admitted patients compared to other studies we discussed. We also used a relatively short duration and narrow age range (18 to 70 years), excluding the pediatric age group. Future studies with larger populations, broader age ranges, and longer duration will help mitigate these limitations.

## Conclusions

Our study showed that the frequency of autonomic dysfunction among patients of Guillain Barre syndrome was significant, consistent with previously done studies as noted in the literature. However, no such study was done in Pakistan to the best of our knowledge. In the present study the most frequent autonomic dysfunctional manifestations were constipation and diarrhea. Our study explored the adverse outcomes of autonomic dysfunction in patients with GBS and enabled increased understanding for physicians so that effective management plans can be formulated for patients with GBS to prevent adverse outcomes.
